# Scimitar Syndrome Incidentally Identified During the Workup for Acute Appendicitis in a Young Adult Female: A Case Report

**DOI:** 10.7759/cureus.101991

**Published:** 2026-01-21

**Authors:** Deep Parkash, Hamed Al-Aamri, Essem Ahmed Rashad Ahmed, Moosa A Alwardi, Hosam M Elghadban, Ayman Albatanony

**Affiliations:** 1 General Surgery, Ibra Hospital, Ministry of Health, Ibra, OMN; 2 Radiology, Ibra Hospital, Ministry of Health, Ibra, OMN

**Keywords:** appendicitis, cardiac dextroposition, horseshoe lung, laparoscopic appendectomy, pulmonary venolobar syndrome, scimitar syndrome

## Abstract

While congenital pulmonary venolobar syndrome (CPVS), including Scimitar syndrome and horseshoe lung, is highly uncommon in adults, acute appendicitis is a common surgical emergency.

This case report emphasizes the difficulties in diagnosing patients and the possibility of misdiagnosis, highlighting the significance of clinical awareness for the best possible care. A laparoscopic appendectomy was performed on a 21-year-old female patient who presented with symptoms of acute appendicitis. During the procedure, a suspicious cecal tumor and a subserosal, dilated retrocecal appendix were observed. The patient was identified as having right lung hypoplasia, cardiac dextroposition, and partial anomalous pulmonary venous return (PAPVR), indicating the presence of Scimitar syndrome along with horseshoe lung. These findings were confirmed by post-operative CT imaging. The appendix histopathology and colonoscopy were unremarkable, and the cardiac assessment, including the electrocardiogram and echocardiogram, was also normal. Incidental CPVS in adults requires diligent recognition for optimal management. This case contributes to the limited research on adult manifestations of Scimitar syndrome and associated abnormalities, which is expanded by this instance.

## Introduction

Congenital pulmonary venolobar syndrome (CPVS) is the umbrella term, characterized by anomalous drainage of the right pulmonary veins into the inferior vena cava (IVC) and frequently associated with right lung hypoplasia and anomalous systemic arterial supply from the aorta. Scimitar syndrome, referring specifically to the subset characterized by anomalous right pulmonary venous drainage, is exceedingly rare [1,2].

Scimitar syndrome is believed to result from an early developmental anomaly of lung bud formation and from compensatory mechanisms leading to an asymptomatic course into adulthood [3].

The “Scimitar syndrome” name comes from the arc-shaped abnormal vein on chest X-rays, resembling a Turkish sword, the Scimitar [4]. Its incidence is 1-3 per 100,000 live births, with a female-to-male ratio of about 2:1. About 90% are diagnosed in infancy due to respiratory issues, infections, or heart failure [5]. Adult cases are rare, usually asymptomatic, and often found incidentally. Many display no symptoms, but when symptoms occur, they include recurrent lung infections and exertional dyspnea [6].

Scimitar syndrome identification relies on imaging, with CT scan and chest X-rays often showing a curved silhouette (Scimitar sign), right lung hypoplasia, and cardiac dextroposition with mediastinal shift. Horseshoe lung fusion is rare and linked to the syndrome [1,3]. CT scan is preferred, but MRI and echocardiography are alternatives when CT scan isn’t suitable. MRI offers detailed vascular and soft-tissue views, while echocardiography assesses heart function. Treatments include observation or surgery, depending on symptoms and anomalies [7].

Acute appendicitis is a common disorder, with a 7%-8% lifetime incidence, making it a major cause of acute abdominal surgery worldwide. In adults, it usually presents straightforwardly [8]. Cases of appendicitis with incidental CPVS and Scimitar syndrome are rare in the literature. Our patient had no cardiopulmonary symptoms, aligning with reports of adult Scimitar syndrome, which is often asymptomatic and found incidentally [1]. Surgeons should consider these conditions and perform thorough evaluations in unusual cases to translate epidemiological data into clinical insights.

The incidental detection of CPVS during evaluation for acute appendicitis represents a clinically important scenario that highlights the value of comprehensive postoperative imaging when intraoperative findings are unexpected. This case contributes to the limited literature on asymptomatic adult presentations of Scimitar syndrome with horseshoe lung identified through surgical pathways.

Our objective is to describe a rare incidental discovery of CPVS (Scimitar syndrome with associated horseshoe lung) in an asymptomatic adult undergoing evaluation for acute appendicitis and to highlight the clinical relevance of comprehensive postoperative imaging when unexpected intraoperative findings arise.

## Case presentation

The patient, a 21-year-old woman, came to the Emergency Department (ED) with severe right lower quadrant abdominal pain, nausea, vomiting, and anorexia for one day. Her gynecologic history included regular monthly periods with no fever noted, and her last period was three days prior to admission. She had no significant past medical history identified.

Her vital signs at the time of evaluation were within the limits of normal, and she had an elevated body temperature of 37.7°C. An examination of the abdomen revealed localized tenderness in the right iliac fossa, along with rebound soreness and a positive Rovsing sign. Acute appendicitis was suggested by the Alvarado score. Leukocytosis with neutrophilia was found in laboratory studies. Beta-human chorionic gonadotropin (β-hCG) was negative, and C-reactive protein (CRP), renal function, electrolytes, and urine microscopy were all within normal ranges. A non-compressible, blind-ended, 9-mm tubular structure with an appendicolith and surrounding mesenteric echogenicity was visible on ultrasound.

**Figure 1:**
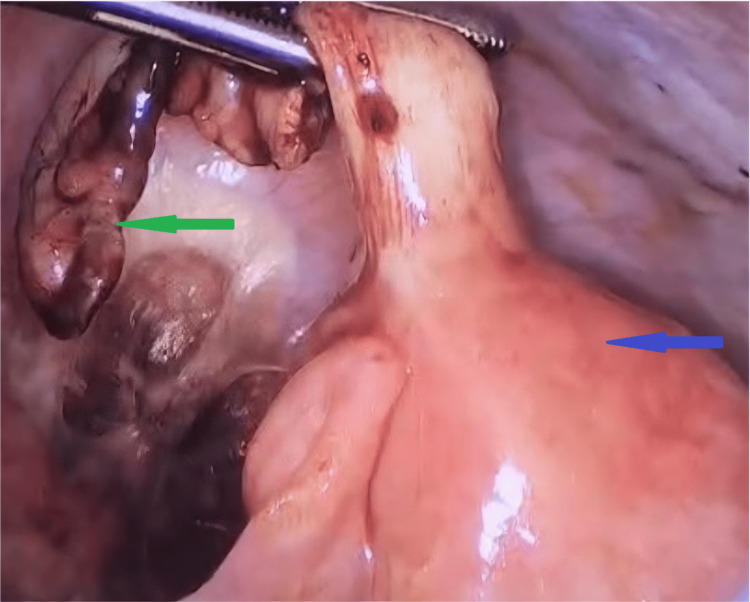
Laparoscopic intraoperative view showing a thickened cecal wall (blue arrow) and an inflamed appendix (green arrow).

A laparoscopic appendectomy was performed on the patient. Intraoperatively, the appendix appeared inflamed, with subserosal distension; no evidence of Meckel's diverticulum was present, and the terminal ileum was found to be grossly normal. However, final histopathological examination revealed no acute inflammation, chronic inflammation, or architectural distortion. This discordance between gross appearance and microscopic findings may represent early or resolving appendicitis, subserosal edema without mucosal involvement, or reactive changes. The clinical presentation (Alvarado score, imaging findings) supported the preoperative diagnosis, and the decision to proceed with appendectomy was appropriate, given the clinical context (Figure 1).

**Figure 2:**
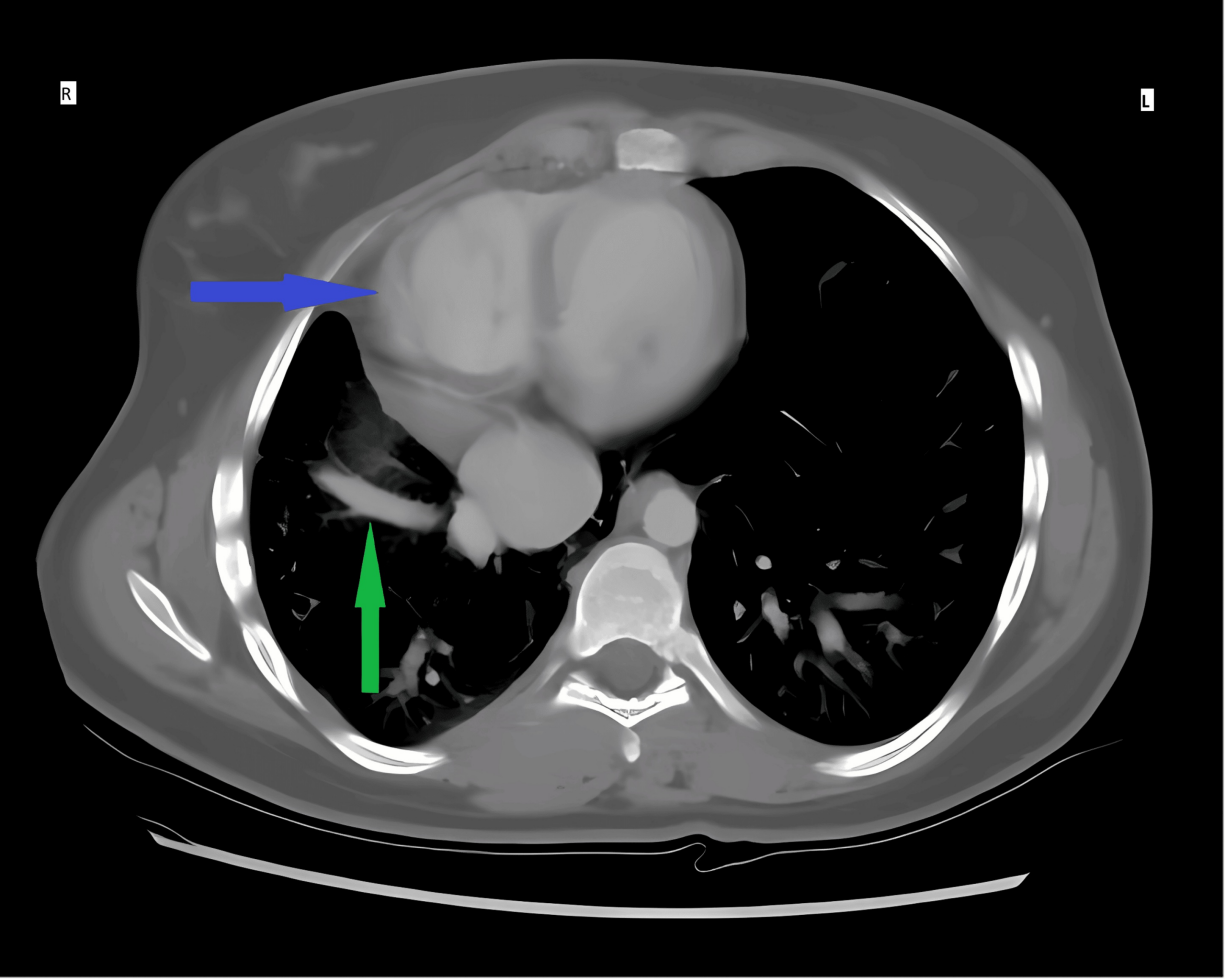
Axial CT scan (mediastinal window) The blue arrow demonstrates the mediastinal and cardiac shift to the right side (cardiac dextroposition), associated with abnormal drainage of the pulmonary veins (green arrow) into a dilated inferior vena cava. Cardiac dextroposition (rightward shift) was identified secondary to right lung hypoplasia, distinct from true dextrocardia, which involves primary cardiac malposition. Labels: R = right lung, L = left lung.

**Figure 3:**
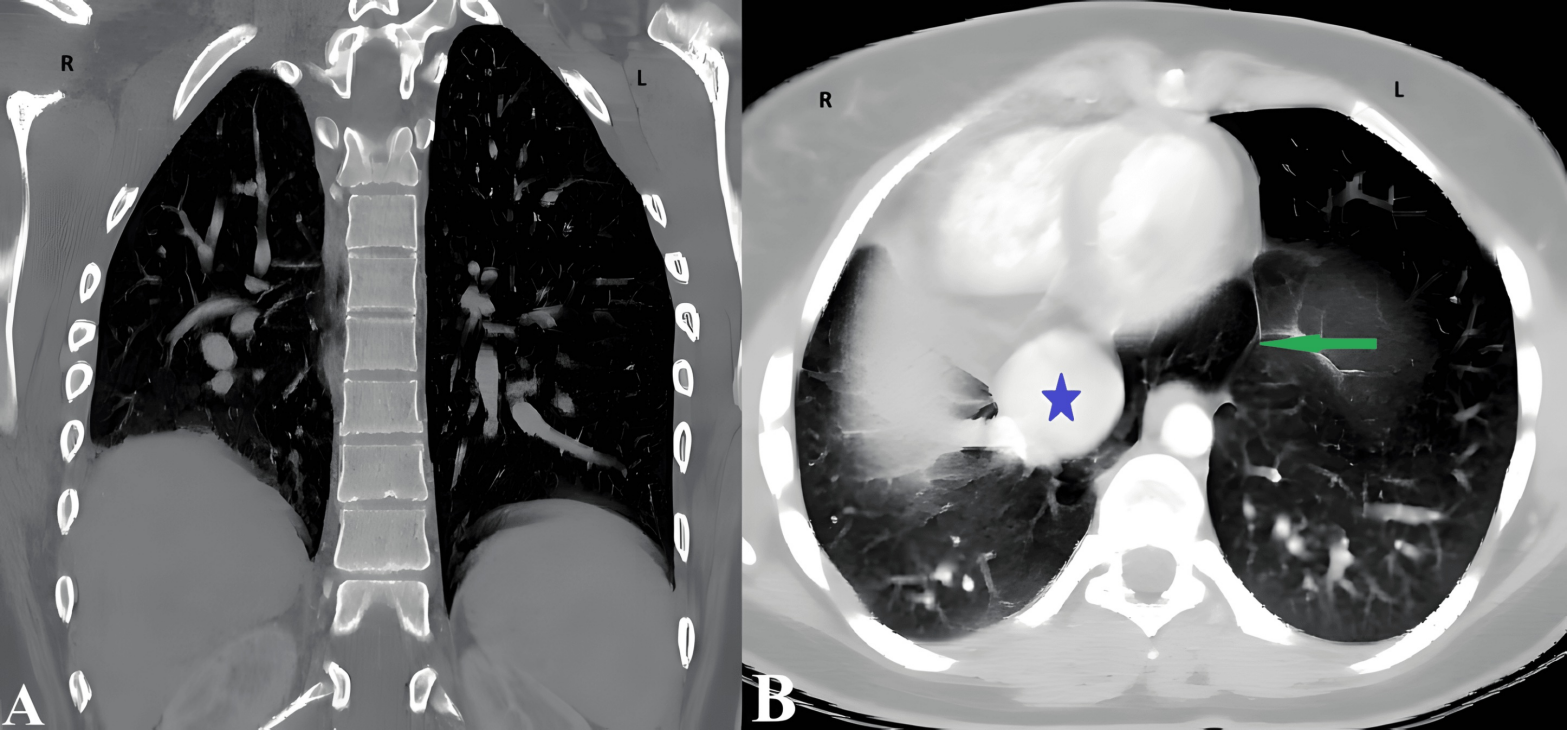
Coronal (A) and axial (B) CT of the chest (lung windows) An overall hypovolemia of the right lung (hypoplastic lung), with compensatory hypervolemia of the left lung. Well-aerated, retro-cardiac, tongue-like projection (green arrow) of the right lung crossing the midline (horseshoe lung), with a dilated, congested inferior vena cava (blue star). Labels: R = right lung, L = left lung.

The patient's post-operative course was unremarkable. In response to the unexpected operative findings, a postoperative abdominal contrast-enhanced CT scan was performed, demonstrating cecal wall thickening with intramural air locules. Additionally, an incidental cardiac dextroposition was found in the superior views, and therefore, a chest CT scan was performed within 24 hours. This rapid diagnostic cascade identified a hypoplastic right lung, cardiac dextroposition, and partial anomalous pulmonary venous return (PAPVR) draining into the IVC (Figure 2), which is radiologically consistent with Scimitar syndrome. A horseshoe lung configuration was also identified (Figure 3). The cardiac evaluation demonstrated no hemodynamic abnormality, and she remained asymptomatic. According to histopathology findings, the appendix was normal and showed no signs of cancer or inflammation. Following an effortless recovery, the patient was discharged with recommendations for cardiopulmonary follow-up. CT scans (with contrast) can demonstrate the typical signs of Scimitar syndrome, as well as the anatomy of an abnormal vein that arises from the upper right lung and drains into the IVC in a curved fashion (“Scimitar” sign). Chest CT scan demonstrated a hypoplastic right lung, cardiac dextroposition, PAPVR draining into the IVC (Scimitar sign), and horseshoe lung configuration. Cardiac evaluation, including ECG and echocardiogram, showed no hemodynamic abnormality. Therefore, CT scan provides a complete anatomical picture necessary to confirm the diagnosis and plan the appropriate surgery.

## Discussion

Scimitar syndrome is part of the broader congenital pulmonary venolobar spectrum and is typically diagnosed in infancy. Adult presentations are rare and most often identified incidentally during imaging for unrelated disorders. The conventional clinical presentation is characterized by a triadic manifestation: a PAPVR, hypoplasia of the right lung, and rightward cardiac shift. Horseshoe lung, a parenchymal fusion of both lungs posterior to the heart, occurs in about a quarter of reported cases. Most patients present early in life with respiratory or cardiac manifestations, whereas adults are frequently asymptomatic, with occasional mild exertional dyspnea, recurrent thoracic infections, or incidental radiological observations [1,3,9]. 

The present case lacked cardiopulmonary symptoms, consistent with the literature, which describes adult Scimitar syndrome as largely subclinical and often discovered only through cross-sectional imaging performed for other reasons [10]. For patients with incidentally discovered CPVS, individualized follow-up plans should be developed in consultation with cardiology and pulmonary specialists, based on the specific anatomic features and hemodynamic significance. Clinicians can respond promptly to symptoms or problems and continue systematic long-term follow-up for asymptomatic people using these regimens. CPVS includes a wide range of developmental abnormalities; several features from this spectrum were observed in the present case and match the established descriptions found in the literature [10].

Horseshoe lung itself is exceedingly rare and is strongly associated with Scimitar syndrome, as demonstrated in multiple case series and radiologic reviews. Contrast-enhanced CT is the imaging modality of choice for CPVS, demonstrating the anomalous venous drainage, associated anatomic variants, and hemodynamic consequences [2,3]. This case reflects that trend, in which postoperative imaging for suspected cecal pathology unexpectedly revealed a complex congenital cardiopulmonary anomaly.

The histopathological finding of a normal appendix, while unexpected, reflects a recognized clinical scenario. Negative appendectomy rates of 6%-15% are generally accepted in surgical practice, balancing the risk of missed perforation against unnecessary surgery [11-15]. This case reinforces that clinical decision-making, based on presentation and imaging findings, remains appropriate even when histopathology is ultimately unremarkable.

This case demonstrates that incidental surgical findings can uncover concealed congenital anomalies. Targeted imaging and multidisciplinary evaluation ensured safe, noninvasive management.

## Conclusions

This case illustrates that comprehensive postoperative imaging can reveal previously unrecognized congenital cardiopulmonary anomalies when intraoperative findings differ from preoperative expectations. While our patient with Scimitar syndrome and horseshoe lung remained asymptomatic and required only conservative management, this case adds to the limited adult literature on incidental CPVS detection.

Multidisciplinary evaluation enabled appropriate risk stratification and follow-up planning. The histopathological finding of a normal appendix, despite clinical and imaging features suggestive of appendicitis, underscores the diagnostic challenges inherent in acute abdominal presentations.
